# Holistic Medicine: Scientific Challenges

**DOI:** 10.1100/tsw.2003.96

**Published:** 2003-11-13

**Authors:** Soren Ventegodt, Niels Jorgen Andersen, Joav Merrick

**Affiliations:** The Quality of Life Research Center, Copenhagen K, Denmark

**Keywords:** human development, holistic medicine, public health, Denmark, Norway, Israel

## Abstract

The field of holistic medicine is in need of a scientific approach. We need holistic medicine — and we even need it to be spiritual to include the depths of human existence — but we need it to be a little less “cosmic” in order to encompass the whole human being. Many important research questions and challenges, empirical as well as theoretical, demand the attention from medical researchers. Like a number of other practitioners and researchers, our group at the Quality of Life Research Center in Denmark together with groups in Norway and Israel are trying to tackle the research challenge by using conceptual frameworks of quality of life. We have suggested that quality of life represents a third influence on health beyond the genetic and traumatic factors so far emphasized by mainstream medicine. In our clinical and research efforts, we attempt to specify what a clinician may do to help patients help themselves, by mobilizing the vast resources hidden in their subjective worlds and existence, in their hopes and dreams, and their will to live. The field of holistic medicine must be upgraded to fully integrate human consciousness, scientifically as well as philosophically. We therefore present a number of important research questions for a consciousness-based holistic medicine. New directions in healthcare are called for and we need a new vision of the future of the healthcare sector in the industrialized countries. Every person seems to have immense potentials for self-healing that we scarcely know how to mobilize. A new holistic medicine must find ways to tackle this key challenge. A healthcare system that could do that successfully would bring quality of life, health, and new ability of functioning to many people.

## INTRODUCTION

The field of holistic medicine has been too preoccupied with orthomolecular medicine and dietary supplements without much scientific foundation. Acupuncture needles and other seemingly effective, but strange, elements from the traditional medicines, semi-religious procedures of meditation and healing manipulating “energetic dimensions” like “chakras” and “auras” in symbolic ways, and similar approaches have been in the forefront, but not easily compatible with the basic reason and common sense of a modern medical science.

We most definitely need holistic medicine — and we even need it to be spiritual to include the depths of human existence — but we need it to be a little less “cosmic” than that expressed by Deepak Chopra[1] and a little more into the scientific world. To encompass the whole human being, a truly holistic medicine needs to include the dimension of consciousness in a scientific way: the subjective experience of the patient and interpretations of the world[2], his/her feelings, hopes and fears, actual sense of coherence[3], level of self-expression or self-actualization [4], and basic perspective of life[5].

Within the past 15 years, consciousness has erupted as a legitimate research topic among neurobiologists[6,7,8,9,10,11]. Medical scientists are beginning to take notice and become aware, and scientific journals (like the *Journal of Consciousness Studies*) are now publishing articles from physicians more often. Needless to say, holistic medicine must equally integrate the influence of personal consciousness on the course, prevention, and treatment of disease.

Formidable research challenges loom, however. Many important research questions, empirical as well as theoretical, demand attention from medical researchers. Like a number of other practitioners and researchers, our group at the Quality of Life Research Center in Denmark together with groups in Norway and Israel are trying to tackle the research challenge. Using conceptual frameworks like quality of life[12,13,14,15], we have suggested that a patient’s quality of life represents a third influence on health beyond the genetic and traumatic factors so far emphasized by mainstream medicine. In our clinical and research efforts, we attempt to specify what a clinician may do to help patients help themselves, by mobilizing the vast resources hidden in their subjective worlds, in their hopes and dreams with their will to live.

## AN INVITATION FOR DEBATE

The field of holistic medicine must be upgraded to fully integrate human consciousness, scientifically as well as philosophically[16]. So as to invite broad international collaboration from medical scientists and other researchers, we present below a number of important research questions/topics for a consciousness-based holistic medicine. These will also be discussed at The First Nordic Conference on Holistic Medicine planned to take place in 2004.

### 1 — Why are Quality of Life, Health, and Functioning so Closely Interlocked?

Recent research in holistic medicine shows that quality of life, health, and ability are strongly correlated statistically. Studies in highly esteemed journals like *the Lancet* point in the direction that patients with cancer and cardiovascular diseases, who improve their quality of life, live longer than patients who do not improve their quality of life[17,18]. Patients who improve their quality of life also seem to have fewer symptoms[19] and to be more able and better related to people close to them.

## 2 — Why is Self-Assessed Health the Most Robust Health Indicator in Town?

Over the past 10 years, it has become evident that the patient knows more about his/her health status than the physician, even after careful examination. Many studies suggest that self-assessed health is the best predictor of survival[20,21,22,23,24,25]. Why is it so important to experience oneself as healthy, and how does this experience create in the patient?

## 3 — Healing of Tissue Damage: Can Degenerated Cartilage Heal?

A surprising number of healings have been documented scientifically[26]. There are also stories of damaged cartilage that has been observed to heal in X-rays. But what does it take for an injured cartilage to heal? Holistic health practitioners have several theories for this, and those theories need testing.

## 4 — Spontaneous Remission of Cancer: Can We Induce It?

Cancer patients deemed terminal by their physician have sometimes managed to rid themselves of their cancers. The Danish physician and scientist Ulrik Dige has described 45 such cases in his book *Cancer Miracles*[27]. In two or three cases, the patient experienced a spiritual opening prior to the healing, a breakthrough to a new understanding of life. Can theories of holistic medicine explain this? And is it possible to induce spontaneous healing in cancer patients? Can we believe the evidence, from sources such as video recordings of Body-Mirror System healing, that tumors practically melt away like snowballs after a few hours of treatment?

## 5 — Health Through Quality-of-Life Improvements and Personal Growth

What is it to engage in personal growth and empty the inner waste basket of painful emotions and repressed truths about ourselves? Can repressed emotions make you sick? Could Louise Hay[28] and others of the controversial health gurus be just a little bit right in spite of their unscientific or even contra-scientific explanations? If so, what does it take to make emotional pain resurface and eventually go away, so the patients regain their health?

## 6 — Personal Resources, Known as Well as Hidden

Deep within us are vast resources, such as joy of life, boundless energy, and intuitive competence. Given half a chance, any patient can access these resources, but they are all too often ignored as modern healthcare systems reduce patients to impassive objects of treatment. Helping a patient upgrade her personal philosophy of life is often very difficult, because negative attitudes to life often derive from a personal history of failure, which has to be confronted and dealt with.

## 7 — Holistic Medicine in General Practice

What can the general practitioner or family physician do if s/he wants to involve a patient in the treatment? How do we help the patient take charge of his or her own life? What is good “holding” — support — for the patient, and how far can we get just by using attention, respect, and care?

## 8 — When the Physician is Ill

The physician is often powerless when s/he becomes ill. Everyone considers this a bit of an anomaly. Out of shame, many doctors try to treat themselves, sometimes in vain. What do sick healthcare professionals need and how may we support them in their personal growth and healing?

## 9 — Consciousness and Brain Function

What is consciousness? How does the brain work 29]? Do our emotions reside in the body? What is the subconscious and where is it located? How do cells create consciousness? How do our decisions and consciousness intervene in the cellular order and make us ill? There are a thousand questions to be answered and a modern holistic theory of what it is to be human being may help us along.

## 10 — Emotional Intelligence: What is It? Can We Develop It?

One recent theory of emotions claims that they are organized in an intelligence radically different from our mental intelligence[30,31]. Traditional IQ is discriminative and analytical, but emotional intelligence (EQ) is intuitive and synthetic, i.e., integrative and holistic. People who develop their EQ become more loving, socially skilled, and sexually well functioning. Leadership and collaborative skills are a matter of EQ as much as IQ.

## 11 — Holistic Psychiatry: Holding and Spontaneous Remission of Schizophrenia

It is well known from the recovery literature that some 20–30% of patients with juvenile schizophrenia regain their sanity. It seems as if recovery sets in when the patient succeeds in finding and bonding with a person who sincerely understands, accepts, and loves him or her. The research question is: can this loving or holding be delivered by the healthcare professional, so that young people with psychological problems may avoid a lifelong career as a psychiatric patient?

## 12 — Immune System Regulation

The immune system's capacity for self-nonself discrimination seems to have very early evolutionary roots[32]. Yet, this capacity seems to be subject to modulation by psychological and existential factors. Thus, there may be psychodynamic explanations behind a number of autoimmune diseases, such as juvenile diabetes, which may prove amenable to treatment by some variety of holistic medicine.

## 13 — A Psychoimmunological Interpretation of HIV

The connections between subjective experience and immune function[33] may possibly be exploited to prevent HIV patients from developing AIDS. Sero-conversion from HIV positive to HIV negative has been known to occur in patients treated through alternative medicine, such as healing. This raises the possibility that holistic medicine may offer treatment based on interventions in the patient's consciousness and subjective experience.

## 14 — What can We Learn from the Healing Systems of Indigenous Cultures?

Traditional medicines in most preindustrial cultures were directed towards human consciousness. The Indian medicine wheel and peyote medicine[34], the African cultures, or the Australian aboriginals all had the shaman or sangoma enter altered states of consciousness to help the patient know himself, so that a series of existential choices would lead to a new life, often as a cured person. The various traditional healing systems share a number of features, which, if integrated into a theory of “primitive” medicines, may explain what happens when a patient is cured through changes in perspective and philosophy of life.

## 15 — Sexuality as an Avenue to Personal Growth

Many paths lead to personal growth, but some are more effective than others. Surprisingly, sexuality is a particularly direct path, as the followers of the late, highly controversial guru Osho have discovered[35]. Any significant problem that a person has will manifest itself in his or her sexuality. It is often easy to recognize what is natural and what is not in one’s sex life. Lifestyle changes in diet, exercise, and drinking habits are hard won, whereas similarly dedicated attempts to make corrections in one’s sex life, alone or with one’s partner, often bear fruit and may lead to dramatic changes in self-assessed physical and psychological health status. If a sound sexuality really is a path to health, we are obliged to overcome the taboos and integrate sexuality into a holistic medical science.

## 16 — Health Economics: Can We Create Cost-Effective Holistic Care?

Holistic medicine is not a cure-all, but its popularity is growing. When patients help themselves by their own lights, instead of relying on expensive public services, quite a bit of money can be saved in the human services and healthcare sectors. How do we know which patients are particularly well suited to holistic treatment? Patients likely to benefit from holistic medicine are those that are ready to take responsibility for life’s emotional pain, for only such patients are strong enough to drop their pretensions and inhibitions, and accept their real selves and become authentic, true, and alive in the present. But how do we know who has this readiness?

## 17 — Quality Assurance and Quality Improvement in Holistic, Alternative, or Complementary Medicine

Severely ill patients seem often to be fooled into spending a fortune on perhaps futile dietary supplements, blood tests, and various pills. How do we clean up and separate the real from the phony? What is quality? How do we counsel our patients? Everything that helps the patient feel, understand, and let go of negative attitudes to life seems to be of value from a theoretical perspective, while procedures that do not lead the patient towards a more positive attitude to life, and a better quality of life, seem to be of less value if not worthless. But can we make the patient stop digesting redundant vitamin and mineral pills and undocumented shark cartilage and get them to use their resources in a more constructive manner? And should we not test all the popular alternative cures for possible beneficial effects, before we judge them to be of no value? And if they have an effect, i.e., only because of the placebo effect, should we not take that seriously and develop more efficient placebo cures for the patient who needs them?

## 18 — Is there Room for Concepts like Life Energy in Medicine?

Ancient practices, such as ayurveda (an intricate system of healing that originated in India thousands of years ago), have employed meditation and energy work to help the patient regain health and wholeness. Modern biochemical medicine has ridiculed the concept of psychological energy and has tried to eradicate the idea of a life force, an élan vital. However, the experience of energy in one's life is closely associated with healing. Personal growth is almost always about becoming more energetic and happy.

## 19 — Research Methodology

Are randomized clinical trials suitable for research in holistic medicine? What are good endpoints in studies of holistic medicine? How is quality rated and how valid is psychometric testing? Most physicians will probably agree that we need clinical trials with "soft" and subjective endpoints, but how should such studies be carried out? What about the many therapeutic effects of the patient-physician relationship that are easily controlled away in these studies because they are not measured? Can the concept of quality of life solve these problems sufficiently?

## 20 — Consciousness, the Hermeneutic Problem and Planted Memories

A patient lives in a self-confirming bubble, just as a scientist or any regular person does. The hermeneutic problem has not been solved, but influential thinkers in philosophy and scientific methodology have taken steps in the right direction[36,37,38,39]. In a consciousness-oriented medicine, this problem has new and unpleasant implications. For how can we know what happens when we make interventions aimed at the patient's consciousness? How do we avoid producing damage, such as planted memories of incest?

## 21 — Is the Consultation Interview the Doctor's Most Important Instrument?

What is a conversation? What are its elements and structure? What skills does it require and how do doctors train them? How do you conduct a conversation about existential matters? How much can you say to a patient? When is a provocation in order? Can you always tell the truth[40]?

## 22 — Meeting Soul to Soul

When two people meet on a deep and soulful level, magic happens. Such a meeting is an invitation to explore the self and begin a journey into life and its potentials. If you can meet another person without reservation and accept him or her exactly as s/he is, a new awareness of talents and purpose in life may result. The person being thus met may well find a completely new direction in life[41].

## 23 — Holistic Touch: Meeting Soul to Soul Through the Body

There are many kinds of touch. The special kind of touching used in holistic therapy invites the patient to become present in a part of the body that is ill or over- or undersensitive or where the patient is just absent[42]. Oftentimes, this invitation to become present, and the associated meeting soul to soul between patient and doctor through the medium of the patient's body, produces a violent emotional reaction in the patient which, from time to time, leads to rapid healing after the patient has confronted the weak body part.

## 24 — Research Policy and Economics: We Urgently Need an Inexpensive Research Paradigm for Evidence-Based Holistic Medicine

The Cochrane standard has made medical research so costly that only the wealthy, medical industry can afford it. Research in holistic medicine, where patients heal themselves by improving their quality of life[19], has no financial interest, so it has become almost impossible to get funding for this promising line of research. We urgently need a new research paradigm for the research in alternative, holistic, and complementary medicine to develop an evidence-based holistic medicine. As this seems to be the most important problem facing the development of holistic medicine, we have made a suggestion for such a new paradigm, "the square curve paradigm", reducing the price of a scientific study of a medical treatment with a factor 100, without compromising the scientific validity[43]. The idea is that an immediate improvement of quality of life, health, and ability of functioning can be demonstrated without the use of a control group. We hope that this new paradigm will be generally accepted by the medical community.

## CONCLUSIONS

It appears that new areas of research like psychoneuroimmunology and life mission theory are opening new vistas in medical research concerned with consciousness, wholeness, and even spirituality, if this latter term can find a meaning in science.

The life mission theory[15] claims that everyone has a deep meaning in life that can be known as a purpose in life, a task that a life may be devoted to carrying out. When patients discover their purpose in life and learn to live accordingly, they often heal and become happier and more valuable to themselves and others. Evidently, such a theory raises more questions than it answers.

New directions in healthcare are called for. We need a new vision of the future of the healthcare sector in the industrialized countries. Every person has immense potentials for self-healing that we scarcely know how to mobilize. A new holistic medicine must find ways to tackle this key challenge. A healthcare system that could do that successfully would bring quality of life, health, and ability of functioning to many people. Are we able to change the healthcare system over the next hundred years to focus on the hidden potentials of man, or do we need a new kind of holistic health centers, complementary to the established biomedical ones, for holistic medicine to be developed and established in our society?

## BIOSKETCHES

**Søren Ventegodt, MD,** is the Director of the Quality of Life Research Center in Copenhagen, Denmark. He is also responsible for a Research Clinic for Holistic Medicine in Copenhagen and is a popular speaker throughout Scandinavia. He has published numerous scientific or popular articles and a number of books on holistic medicine, quality of life, and quality of working life. His most important scientific contributions are the comprehensive SEQOL questionnaire, the very short QoL5 questionnaire, the integrated QOL theory, the holistic process theory, the life mission theory, and the Danish Quality of Life Research Survey, 1991–94 in cooperation with the University Hospital of Copenhagen and the late pediatric professor Bengt Zachau-Christiansen. E-mail: ventegodt@livskvalitet.org. Website: www.livskvalitet.org/

**Niels Jørgen Andersen, MSc,** Professor, Department of Innovation and Economic Organization, Norwegian School of Management. This department conducts research and provides teaching in central topics related to innovation, business development, management of global companies, business history, and economic organization. Research activities within the Department are related to four core subjects within the discipline: business history, cooperative organizations, business development and entrepreneurship, and finally studies of industries with a special focus on the electricity industry. He is also the dynamic chairman of the nonprofit organization Stiftelsen Holistisk Medisin Scandinavia, which aims to support the scientific development, research, and documentation of complementary and holistic medicine in Scandinavia. E-mail: niels.j.andersen@bi.no. Website: www.bi.no/users/fgl93013/

**Joav Merrick, MD, DMSc,** is Professor of Child Health and Human Development affiliated with the Zusman Child Development Center and Division of Community Health at the Ben Gurion University, Beer-Sheva, Israel and presently the Medical Director of the Division for Mental Retardation, Ministry of Social Affairs, Jerusalem and the Director of the National Institute of Child Health and Human Development. He has numerous publications in the field of child and human development, rehabilitation, intellectual disability, disability, health, welfare, abuse, advocacy and prevention. Dr. Merrick received the Peter Sabroe Child Award for outstanding work on behalf of Danish Children in 1985 and the International LEGO-Prize ("The Children's Nobel Prize") for an extraordinary contribution towards improvement in child welfare and well being in 1987. E-mail: jmerrick@internet-zahav.net. Website: www.nichd-israel.com
